# Bioactivity of *Artemisia dracunculus* and *A. absinthium* Essential Oils Against Stored Product Insect Pests and Root-Knot Nematodes

**DOI:** 10.3390/insects17080837

**Published:** 2026-08-12

**Authors:** Emre Evlice, Mustafa Alkan, Ömer Cem Karakoç, Sait Ertürk, Reyhan Bahtiyarca, Yasemin Yücel Yücel, Ali Rıza Tüfekçi, Ümit Yırtıcı

**Affiliations:** 1 Department of Plant Protection, Faculty of Agricultural Sciences and Technology, Sivas University of Science and Technology, 58000 Sivas, Türkiye; emre.evlice@sivas.edu.tr; 2 Department of Plant Protection, Faculty of Agriculture, Yozgat Bozok University, 66900 Yozgat, Türkiye; 3 Vocational School, Çankırı Karatekin University, 18100 Çankırı, Türkiye; 4 Department of Plant Pest, Directorate of Plant Protection Central Research Institute, 06172 Ankara, Türkiye; sait.erturk@tarimorman.gov.tr; 5 Field Crops Central Research Institute, 06170 Ankara, Türkiye; 6 Department of Biochemistry, Faculty of Pharmacy, Altinbas University, 34145 İstanbul, Türkiye; 7 Natural Products Research and Development Center, DÜAGEM, Altınbaş University, 34145 İstanbul, Türkiye; 8 Department of Chemistry, Faculty of Natural Science, Çankırı Karatekin University, 18100 Çankırı, Türkiye; areb@karatekin.edu.tr; 9 Department of Medical Laboratory, Health Services Vocational School, Kırıkkale University, 71450 Kırıkkale, Türkiye

**Keywords:** biopesticides, contact toxicity, repellency, nematicidal activity, SDG 2

## Abstract

Investigating alternatives to synthetic chemicals in pest management is essential. The bioactivity of *Artemisia absinthium* and *A. dracunculus* essential oils (EOs) was evaluated against two stored-product insect pests (*Sitophilus granaries* and *Tribolium castaneum*) and two root-knot nematodes (*Meloidogyne incognita* and *M. chitwoodi*). EOs were hydrodistilled and analyzed by GC-MS: *A. absinthium* consisted mainly of myrtenyl acetate (47.10%) and β-thujone (11.41%); *A. dracunculus* of methyleugenol (26.02%), β-asarone (14.04%), and elemicin (10.95%). In contact bioassays, *A. absinthium* caused 93.2–96.8% mortality against *S. granarius*, but minimal effect on *T. castaneum*. *A. dracunculus* caused 66.3–73.5% mortality against *S. granarius*, no effect on *T. castaneum*, yet reduced progeny by 87%. Both EOs strongly repelled *T. castaneum* but weakly affected *S. granarius*. *A. dracunculus* exhibited 100% nematicidal efficacy against *M. incognita* and 94.9% against *M. chitwoodi*; *A. absinthium* showed limited effect. *A. dracunculus* showed minimal hatching inhibition (<30%), whereas *A. absinthium* increased hatching. Sparse Partial Least Squares (sPLS) prioritized pulegone, methyleugenol, β-asarone, β-thujone, myrtenyl acetate, and β-phellandrene as constituents co-varying with AChE/BChE responses, with docking identifying pulegone as the top ligand. These findings demonstrate species-specific bioactivity patterns: *A. dracunculus* shows strong nematicidal and reproductive inhibitory effects, whereas *A. absinthium* shows potent acute toxicity and repellency against *S. granarius*, supporting multi-target biopesticide strategies to mitigate risks of synthetic chemical treatments.

## Simple Summary

Stored grains and growing crops are threatened by insects and plant-parasitic roundworms, and their control often depends heavily on synthetic chemicals. This study examined whether essential oils obtained from *Artemisia absinthium* and *Artemisia dracunculus* could provide safer plant-based alternatives. The oils were tested against two important stored-product insects and two root-knot nematodes. *Artemisia absinthium* oil caused high mortality in the granary weevil but showed only limited activity against the red flour beetle. *Artemisia dracunculus* oil was less toxic to the granary weevil but strongly reduced offspring production and showed very high activity against both root-knot nematode species. Both oils also repelled the red flour beetle. Chemical analysis indicated that several naturally occurring compounds were associated with these biological effects. Overall, the two oils showed different but complementary activities: one was more effective against stored-grain insects, while the other was more effective against nematodes and insect reproduction. These findings suggest that plant essential oils may contribute to the development of more sustainable pest-management tools and help reduce reliance on conventional synthetic pesticides.

## 1. Introduction

The use of synthetic insecticides and nematicides has long been the primary approach for controlling major agricultural pests such as stored-product insects and root-knot nematodes [1,2]. However, widespread chemical use raises serious concerns regarding environmental sustainability, pest resistance development, negative impacts on non-target organisms, ecological disruption, and risks to human health through exposure and residue accumulation [3,4]. These drawbacks highlight the urgent need for eco-friendly, sustainable pest management alternatives such as plant-derived essential oils (EOs).

Plant-derived essential oils (EOs) have gained considerable attention as promising biopesticides. EOs, naturally occurring volatile secondary metabolites from aromatic plants, exhibit a wide spectrum of biological activities, including insecticidal, nematicidal, repellent, antifeedant, oviposition deterrent, inhibition of egg hatching, growth regulatory, and antivector effects [5,6]. Due to their complex composition, EOs can act on multiple biological targets simultaneously, reducing the likelihood of resistance development [5]. Moreover, their effects are often species-specific, making them attractive candidates for integrated pest management [7].

Among plant families, Asteraceae is one of the most extensively studied for its nematicidal and insecticidal properties. Numerous species within this family are known to contain bioactive secondary metabolites with significant potential for managing plant-parasitic nematodes and insect pests [8,9]. However, information regarding the chemical composition and efficacy of *Artemisia absinthium* L. (Asteraceae) and *Artemisa dracunculus* L. (Asteraceae) EOs against key agricultural pests, including *Sitophilus granaries* (Linnaeus, 1758) (Coleoptera: Dryophthoridae), *Tribolium castaneum* (Herbst, 1797) (Coleoptera: Tenebrionidae), *Meloidogyne incognita* (Kofoid & White, 1919) Chitwood, 1949 (Tylenchida: Meloidogynidae), and *Meloidogyne chitwoodi* Golden, O’Bannon, Santo & Finley, 1980 (Tylenchida: Meloidogynidae), remains fragmented and insufficient.

Infestation and deterioration of stored agricultural products by insect pests have been a major concern since the beginning of grain storage practices, causing significant quantitative and qualitative losses. These damages not only lead to the spoilage of grains and their derivatives but also exacerbate food security challenges, particularly in developing countries [10]. According to the Food and Agriculture Organization of the United Nations (FAO), approximately 17% of global food production is lost during storage, with insect pests alone responsible for nearly 10% of these losses, while rodents, mites, and microbial pathogens contribute an additional 7% [11]. Among the most economically important stored-product pests are the wheat weevil, *Sitophilus granarius*, and the red flour beetle, *Tribolium castaneum*. *Sitophilus granarius* significantly reduces the quantity and quality of stored products through its feeding behavior, where adults bore holes into grains and larvae consume the grain contents, resulting in contamination and spoilage [12,13,14]. Likewise, *T. castaneum* is recognized as a highly destructive secondary pest that infests a wide range of stored food products, causing up to 77% physical damage and producing toxic quinones that compromise product quality and render them unfit for human consumption [15,16,17].

In addition to stored-product insects, plant-parasitic nematodes, particularly root-knot nematodes (*Meloidogyne* spp.), represent one of the most devastating threats to global crop production. Recent estimates indicate that these soil-borne pathogens cause annual economic losses exceeding USD 157 billion, accounting for approximately 12.3% of total global production losses in susceptible crops [18]. Among the more than 100 described species within the genus *Meloidogyne*, *M. incognita* is recognized as the most polyphagous and damaging species, often leading to yield reductions ranging from 10% to 85% in horticultural crops [19,20,21]. Furthermore, *M. chitwoodi*, the Columbia root-knot nematode, has emerged as a major quarantine pest, especially in potato production, where internal and external tuber defects can cause 100% loss in market value [22]. The management of these soil-borne pathogens is exceptionally challenging due to their wide host range and the withdrawal of several highly effective but environmentally hazardous synthetic nematicides [23]. Consequently, developing bio-rational strategies, such as the use of plant-derived EOs, is essential to mitigate the impact of *M. incognita* and *M. chitwoodi* in modern agriculture [6,24].

The present study aims to evaluate the insecticidal and nematicidal activities, along with the chemical compositions, of EOs derived from *A. dracunculus* and *A. absinthium* against *S. granarius*, *T. castaneum*, *M. incognita*, and *M. chitwoodi*. The study focuses on assessing their fumigant, contact, repellent, progeny inhibition, and ovicidal effects, as well as their acetylcholinesterase (AChE) and butyrylcholinesterase (BChE) inhibitory activities, and their impacts on second-stage juvenile (J2) mortality and egg hatching under laboratory conditions.

## 2. Materials and Methods

### 2.1. Plant Material Collection and Essential Oils Extraction

*Artemisia absinthium* and *A. dracunculus* plants cultivated in the experimental fields of the Field Crops Research Institute (Ankara, Türkiye) were harvested during their flowering period in May and June 2018. Taxonomic confirmation of both species was performed, and herbarium specimens were archived at the Herbarium of Tokat Gaziosmanpaşa University, Türkiye, under accession codes GOPU 2018 (*A. absinthium*) and GOPU 4106 (*A. dracunculus*). Following harvest, the aerial parts of the plants (flowers and leaves) were separated and dried naturally in a shaded area. For EO extraction, the dried plant material (100 g) was roughly ground and subjected to hydrodistillation for three hours using a Clevenger-type distillation unit. The condenser was kept at a stable 4 °C by connecting it to a Mikrohil recirculating chiller to ensure optimal condensation efficiency. EO yield was expressed as milliliters of oil obtained per 100 g of dry plant material (*v*/*w*). The extracted oils were subsequently dried using anhydrous sodium sulfate, placed into dark glass vials to protect them from light, and stored at −20 °C until they were analyzed for their chemical composition.

### 2.2. Chemical Analysis

To determine the chemical composition of EOs, both gas chromatography with flame ionization detection (GC/FID) and gas chromatography–mass spectrometry (GC/MS) techniques were employed simultaneously. The GC/MS analyses were carried out using an Innowax FSC capillary column (60 m in length, 0.25 mm internal diameter, and 0.25 µm film thickness Agilent Technologies, Wilmington, DE, USA), with helium serving as the carrier gas at a constant flow rate of 1 mL/min. The oven temperature program began with an initial hold at 60 °C for 10 min, followed by an increase to 220 °C at a rate of 4 °C per minute, maintained for an additional 10 min, and finally raised to 240 °C at a slower rate of 1 °C per minute. Mass spectral data were obtained under electron impact ionization at 70 eV, scanning the mass range of 35 to 450 m/z. GC/FID analysis was conducted under identical chromatographic conditions, using the same column, carrier gas, and temperature program, with the detector maintained at 300 °C. Identification of the detected compounds was accomplished by comparing their mass spectra and retention times with entries in commercial libraries (Wiley GC/MS Library and MassFinder 3 Library) as well as an in-house database containing spectra of known reference EOs. Retention indices (RI) were determined by analyzing a standard mixture of n-alkanes and comparing the values with previously reported RI data obtained under similar analytical conditions [25].

### 2.3. Rearing of Tribolium castaneum and Sitophilus granarius

Adult *Tribolium castaneum* were reared on a mixture of ground wheat (*Triticum aestivum* L.) and dry yeast at a ratio of 20:1. The insect colony was maintained under constant laboratory conditions of 25 °C and 60 ± 5% relative humidity. Adult *Sitophilus granarius* were reared exclusively on whole soft wheat grains under the same environmental conditions. Unsexed adults, 7–28 days old, were used in all experiments [26].

### 2.4. Rearing of Meloidogyne chitwoodi and Meloidogyne incognita

In this study, *Meloidogyne chitwoodi* and *M. incognita* populations, which are already maintained as pure cultures in the Plant Protection Central Research Institute, Nematology Laboratory, were used. Egg masses of each population were separately inoculated for each nematode population on tomato plants (Tueza F1). The plants were grown in a climate-controlled chamber at 23 ± 2 °C and harvested eight weeks after planting. To obtain second-stage juveniles (J2s) of *M. incognita* and *M. chitwoodi,* egg masses of either species were placed on sieves (5 cm diameter) positioned inside Petri dishes (12 cm diameter). Enough water was added to cover the egg masses completely, and egg masses were incubated at 25 ± 1 °C. Every 24 h, the newly hatched J2s were collected and stored at 4 °C until use. The number of J2s was counted under a light microscope (Leica DM4000 B LED, Leica, Wetzlar, Germany), and the suspension was adjusted to a final concentration of 100 J2/mL. Only 24–72 h aged J2s were used in bioassays [27].

### 2.5. Test of Contact Activity of Essential Oils Against Insects

The insecticidal activity of EOs was evaluated through a direct contact method, where a 1 µL aliquot of a 10% EO solution (dissolved in acetone) was carefully applied to the dorsal surface of each adult insect using a precision micro-applicator [28]. Acetone served as the negative control, whereas K-Obiol^®^ EC 25 (Bayer AG, Leverkusen, Germany), a commercial product containing 25 g/L deltamethrin and 250 g/L piperonyl butoxide, served as the positive control. Each experimental group, including the controls, consisted of 20 adult individuals per replicate. After treatment, the insects were placed into 90 mm plastic Petri dishes containing 10 g of wheat. All dishes were kept under laboratory conditions set at 25 ± 2 °C with 60 ± 5% relative humidity. Mortality rates were recorded after 24, 48, and 72 h. Insects were considered dead when no movement of the legs or antennae was observed [29]. The entire experiment was arranged using a completely randomized design (CRD) with three replicates for each treatment.

### 2.6. Test of Progeny Inhibition of Tribolium castaneum and Sitophilus granarius

Following the completion of the contact toxicity experiment, all surviving and deceased insects were carefully removed from the treatment containers to eliminate the possibility of further egg-laying or disturbance in offspring development. The remaining wheat within each container was maintained under constant laboratory conditions of 25 ± 2 °C and 65 ± 5% relative humidity, allowing for the natural development of the F_1_ generation. No additional insects were introduced throughout this period [26]. Sixty days after the initial treatment, the total number of newly emerged F_1_ adults was recorded to evaluate the reproductive suppression effects of the EOs. The inhibition rate (IR) was determined using the following equation:

IR (%) = [(Cn − Tn)/Cn] × 100

where IR denotes the percentage reduction in adult emergence, Cn corresponds to the number of adults that emerged in the control group, and Tn represents the number in the treated group.

### 2.7. Test of Repellent Activity of Essential Oils Against Insects

The repellent properties of the EOs were evaluated using a modified version of the area preference assay [30]. Circular filter papers (Whatman No. 1, 9 cm in diameter) were cut in half. One half received 500 µL of acetone to serve as the control, while the other half was treated with 500 µL of EO solutions at concentrations of 1%, 2.5%, and 5% (*v*/*v*) prepared in acetone. After applying the treatments, both halves were rejoined to form a complete disc and placed inside a 9 cm Petri dish. To allow the solvent to fully evaporate, the discs were left exposed to air for five minutes. Subsequently, 20 unsexed, 7–28 days old healthy adult insects (*T. castaneum* or *S. granarius*) were introduced at the center of each dish. The dishes were immediately sealed and maintained in complete darkness under controlled conditions of 25 ± 2 °C and 65 ± 5% relative humidity. The positions of the insects on the treated and control halves of the filter paper were recorded after 2, 4, 8, and 12 h of exposure.

Repellency percentages were calculated based on the following equation proposed [31]:

PR = [(Nc − Nt)/(Nc + Nt)] × 100

where PR represents the repellency percentage, Nc is the number of insects present on the control half, and Nt is the number on the treated half. The repellency effect was further classified according to the five-point rating system [32]. The experimental setup followed a CRD with six replicates per treatment.

### 2.8. Test of Acetylcholinesterase (AChE) and Butyrylcholinesterase (BChE) Inhibition

The inhibitory potential of the EOs on cholinergic enzymes was examined using a modified version of the spectrophotometric technique [33]. Both acetylcholinesterase (AChE) and butyrylcholinesterase (BChE) activities were assessed under identical experimental conditions, employing acetylthiocholine iodide (AChI) and butyrylthiocholine iodide (BuChI) as specific substrates for each enzyme, respectively. EO stock solutions, along with the positive control galanthamine hydrobromide, were prepared in methanol at a concentration of 10 mg/mL, with care taken to ensure that the final methanol concentration within the assay mixture did not exceed 10% (*v*/*v*).

Enzyme assays were performed in quartz cuvettes, with the reaction mixture consisting of 240 µL of DTNB (5,5′-dithiobis-(2-nitrobenzoic acid), 0.125 mM), 192 µL of either AChI or BuChI substrate (0.2 mM), and 1200 µL of Tris-HCl buffer (100 mM, pH 8.0). To this mixture, 20 µL of the test EO solution, the galanthamine positive control, or Tris buffer (blank control) was added. The enzymatic reaction commenced following the addition of 348 µL of the AChE or BChE enzyme solution at a concentration of 0.032 U/mL. Absorbance readings were taken at 412 nm over a 2 min period using a Cary 60 Single Beam UV-Vis spectrophotometer (Agilent Technologies, USA). EO inhibitory activity was tested at concentrations ranging from 0.1 to 10 mg/mL.

The percentage of enzyme inhibition was calculated based on absorbance values recorded during the linear phase of the reaction, within the first 60 s, according to the following equation:

Inhibition (%) = (1 − At/Ac) × 100

where At represents the absorbance change observed in the presence of the EO, and Ac corresponds to the absorbance change in the blank control. Each experimental condition was performed in triplicate.

### 2.9. Nematicidal Effect of Essential Oils Against Meloidogyne Juveniles

Stock solutions of EOs were prepared by diluting in methanol, whereas working solutions were diluted in distilled water with Tween-20. Bioassays were conducted using 16-well culture plates. For single-dose experiments, 990 µL of distilled water containing approximately 120–140 J2 individuals was added to each well. Subsequently, 10 µL of the EO solution was added to each well, resulting in a final concentration of 2 µL/mL for the test treatment. The final concentrations of methanol and Tween-20 in each well did not exceed 1% and 0.3% *v*/*v*, respectively. Methanol, Tween-20, and distilled water solution were used as the negative controls [24].

To minimize evaporation, the plates were sealed with plastic film and wrapped in aluminum foil to ensure complete darkness. The plates were then incubated at 25 °C. After 72 h of exposure, the viability of the nematodes was assessed under an inverted microscope (Leica DMI 400B). Juveniles that displayed a straight, elongated body posture or exhibited slight curvature at the head and tail but remained motionless even upon mechanical stimulation were considered paralyzed. To differentiate between paralyzed and dead individuals, all nematodes were transferred to distilled water for an additional 24 h recovery period. Nematodes that failed to regain motility after this period were classified as dead [34].

EOs exhibiting greater than 90% mortality at the single-dose assay were subjected to additional dose–response assays using concentrations of 2, 1, 0.5, 0.25, and 0.125 µL/mL, following the same bioassay protocol. All experiments were conducted in a CRD with four replicates per treatment and were independently repeated three times.

### 2.10. Test to Effect of Essential Oils on Egg Hatching of Meloidogyne spp.

The egg suspension was prepared to contain approximately 100 eggs per 10 µL of distilled water [35]. For the bioassays, 96-well plates were used. In the single-dose trials, eggs extracted from *M. chitwoodi* and *M. incognita* were dispensed into each well by adding 99 µL of the prepared egg suspension (~100 eggs) followed by 1 µL of EO stock solutions, which were prepared as outlined in Section 2.9. A final EO concentration of 2 µL/mL was applied for the single-dose tests. Methanol, Tween-20, and distilled water solutions were used as the negative controls.

To prevent evaporation, plates were sealed with plastic film, wrapped in aluminum foil to maintain darkness, and incubated at 25 °C. The number of hatched second-stage juveniles (J2s) was recorded daily over a period of three consecutive days, beginning 24 h after incubation, using an inverted microscope (Leica DMI 400B, Leica, Wetzlar, Germany). The inhibitory effect of EOs on egg hatching was calculated according to the following formula [24]:

Corrected hatching inhibition % = [1 − (m_treatment_/m_control_)] × 100.

The experiment followed a CRD with six replicates per treatment and was independently repeated twice.

### 2.11. Sparse Partial Least Squares (sPLS) Regression Analysis

A separate sparse partial least squares (sPLS) regression model was built for each species. The X matrix comprised GC-MS-identified EO constituents, whereas the Y matrix comprised all experimental endpoints reported in the study (Supplementary Table S1). Prior to modelling, variables were mean-centered and unit-variance scaled. Analyses were performed in R version 4.5.1 using the mixOmics framework [36]. The optimal number of latent components was selected using leave-one-out cross-validation by maximizing the mean Q^2^ across response variables; two components were retained for both species.

### 2.12. Molecular Docking

Docking analyses were conducted using the tacrine-bound human cholinesterase crystal structures 7XN1 (AChE–tacrine) [37] and 4BDS (BChE–tacrine) [38] using Schrödinger Maestro v12.8 [39]. Protein structures were prepared with the Schrödinger Protein Preparation Wizard (addition of hydrogens, assignment of protonation states, and structure optimization) [40]. Ligands were prepared using LigPrep at pH 7.0 [38,39]. Docking was performed with Glide in SP mode, and poses were generated and ranked using GlideScore [41]. Energy minimization and related preparation steps used the OPLS4 force field [42]. Grid centers were set based on the co-crystallized tacrine coordinates: for 7XN1, x = 47.84, y = −40.42, z = −30.04; and for 4BDS, x = 133.12, y = 116.1, z = 41.01. For protocol validation, tacrine was redocked into each receptor, yielding RMSD values of 0.386 Å for 7XN1 and 0.245 Å for 4BDS. These RMSD values indicate that the co-crystal binding pose was reliably reproduced [41].

### 2.13. Statistical Analysis

The test results obtained from the bioassays were initially expressed as percentages and then subjected to arcsine square root transformation to improve data normality. The transformed dataset was analyzed through a one-way analysis of variance (ANOVA). Where significant effects were observed, treatment means were compared using Tukey’s post hoc test, with statistical significance considered at the *p* < 0.05 threshold. All statistical computations were carried out using Minitab statistical software, version 18 [43].

## 3. Results

### 3.1. Essential Oil Composition of Artemisia absinthium and Artemisia dracunculus

The GC-MS analysis of the *A. absinthium* EO identified 66 different compounds, representing 95.47% of the total oil composition. The principal constituents were myrtenyl acetate (47.10%), *β*-thujone (11.41%), and pulegone (8.50%). In the EO of *A. dracunculus*, a total of 69 compounds were identified, representing 98.72% of the overall oil content. The dominant components included methyleugenol (26.02%), β-asarone (14.04%), elemicin (10.95%), 2,3,4-trimethoxybenzyl alcohol (3.95%), and citronellol acetate (2.65%) (Table 1).

### 3.2. Contact Toxicity of Essential Oils Against Insects

The EOs evaluated in this study displayed differential contact toxicities against the targeted insect pests. The EO from *A. absinthium* exhibited limited contact efficacy against *T. castaneum*, with mortality rates remaining low and showing only a slight increase from 2.5% at 24 h to 5.4% at 72 h post-application. In contrast, the EO of *A. dracunculus* was entirely ineffective against *T. castaneum*, failing to induce any observable mortality throughout the assessment intervals.

Conversely, both EOs exhibited significantly higher, time-dependent toxicity against *S. granarius*. Among them, *A. absinthium* EO was the most effective, achieving 93.2% mortality within 24 h and reaching 96.8% by 72 h. While *A. dracunculus* EO also demonstrated insecticidal activity against *S. granarius*, its effect was more moderate, with mortality rising from 66.3% at 24 h to 73.5% after 72 h of exposure (Table 2).

### 3.3. Repellency Effect of Essential Oils

Both EOs demonstrated considerable repellent activity against *T. castaneum*, with the degree of repellency being highly dependent on both concentration and exposure time. The EO of *A. absinthium* at a concentration of 5.0% demonstrated significant repellency, categorized as Class IV–V, with a 94.00% efficacy recorded after 2 h. This treatment reached 100.0% repellency (Class V) between 4 and 8 h post-application and remained high (90.00%, Class V) even after 12 h. Lower concentrations of *A. absinthium* also showed substantial repellent effects. At a 1.0% concentration, repellency varied from 88.00% (Class V) at 2 h to 92.00% (Class V) at 4 h, reaching a maximum of 100.0% (Class V) after 8 h, before decreasing to 46.00% (Class III) by 12 h. A comparable trend was noted for the 2.5% concentration, with repellency values consistently exceeding 96.00% during the initial 8 h, subsequently declining to 56.00% (Class III) at the 12 h interval.

*Artemisia dracunculus* EO also showed a strong and persistent repellent effect similar to *A. absinthium*. At 5.0%, repellency reached 80.00% (Class V) after 2 h, increasing to 98.00% (Class V) at 4 h, and maintaining 100.0% (Class V) at 8 h. Notably, repellency remained high at 94.00% (Class V) even after 12 h of exposure. At 2.5% concentration, repellency ranged from 86.00% to 96.00% (all classified as Class V) over the first 8 h, and 84.00% (Class V) was recorded at 12 h. Interestingly, *A. dracunculus* consistently maintained its Class V repellency throughout the experiment, even at the lowest concentration (1.0%), with its effect reaching 100.0% in 8 h and decreasing to 64.00% in 12 h (Table 3).

The repellent activity of both *A. absinthium* and *A. dracunculus* EOs against *S. granarius* varied considerably depending on concentration and exposure duration. The highest repellency for *A. absinthium* was observed at a concentration of 5.0%, with repellency rates of 74.00% (Class IV) at 2 h, 72.00% (Class IV) at 4 h, and 80.00% (Class IV) at 8 h post-treatment. Although repellency decreased to 50.00% (Class III) after 12 h, the EO still demonstrated notable efficacy at this time compared to the other treatments. At lower concentrations (1.0% and 2.5%), *A. absinthium* showed moderate initial activity; however, these effects were less persistent, with the 1.0% treatment failing to record any measurable repellency after 12 h.

Compared to *A. absinthium*, the *A. dracunculus* EO exhibited substantially lower and more variable repellent behavior against *S. granarius*. Even at the highest concentration (5.0%), the initial repellency was limited to 38.00% (Class II) at 2 h, though it showed a peculiar upward trend to 66.00% (Class III) after 12 h. The lower concentrations of *A. dracunculus* (1.0% and 2.5%) failed to provide sustained protection, with repellency values often fluctuating below 40% and reaching negligible levels by the end of the 12 h interval (Table 4).

### 3.4. Progeny Production Inhibition of Tribolium castaneum and Sitophilus granarius

**Figure 1:**
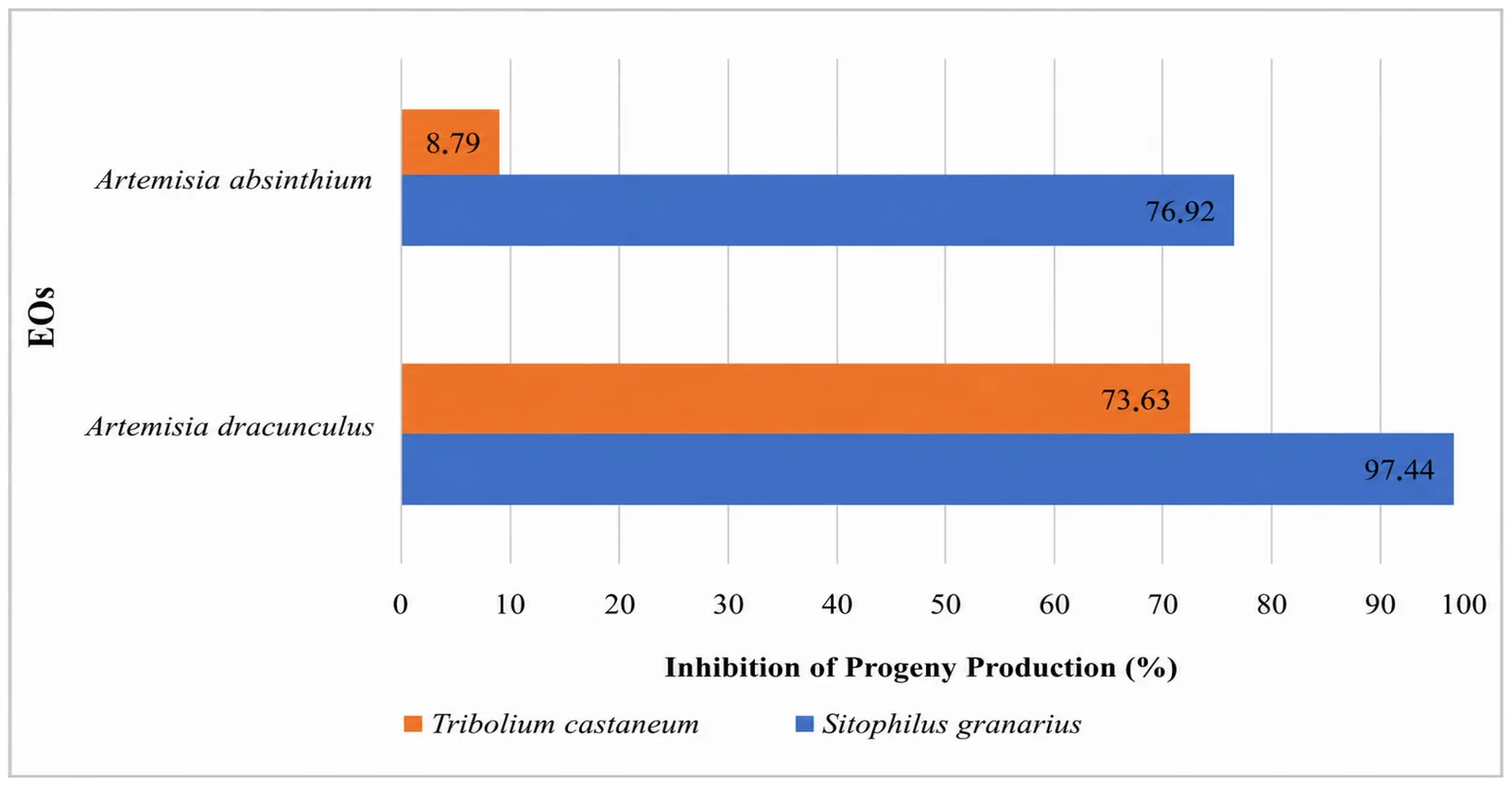
Effect of *Artemisia absinthium* and *Artemisia dracunculus* essential oils (10%) on F1 progeny production in *Sitophilus granarius* and *Tribolium castaneum.*

At the applied concentration, the *A. absinthium* EO exhibited a limited inhibitory effect on F_1_ progeny of *T. castaneum*, achieving only an 8.79% reduction in adult emergence. Conversely, the *A. dracunculus* EO demonstrated a strong suppressive effect, reducing F_1_ adult emergence by 73.63%. When evaluated against *S. granarius*, both oils showed substantial inhibitory activity; *A. absinthium* induced a 76.92% reduction, while *A. dracunculus* reached a near-total suppression rate of 97.44% (Figure 1).

### 3.5. Effectst of Acetylcholinesterase (AChE) and Butyrylcholinesterase (BChE) Inhibition

To evaluate whether the observed insecticidal activity was linked to the disruption of cholinergic neurotransmission, the inhibitory effects of *A. absinthium* and *A. dracunculus* EOs on AChE and BChE were determined. The results indicated that *A. absinthium* exerted a relatively weak inhibitory effect on AChE (14.6%), whereas *A. dracunculus* manifested a markedly higher potency, achieving an inhibition rate of 47.6%. In terms of BChE inhibition, both EOs demonstrated more pronounced activity compared to their effects on AChE. *Artemisia absinthium* showed a moderate inhibitory level (55.2%), while *A. dracunculus* displayed a higher inhibitory effect, reaching 67.7% (Table 5).

### 3.6. Nematicidal Effect of Essential Oils Against Meloidogyne Juveniles

*Artemisia dracunculus* EO induced a mortality rate of 20.3% in *M. incognita* at a concentration of 2 µL/mL within 24 h, which significantly escalated to 86.6% after 48 h and resulted in 100% mortality by 72 h. In contrast, *A. absinthium* exhibited a weak nematicidal effect, with mortality values of 1.2%, 6.4%, and 11.4% recorded at 24, 48, and 72 h, respectively. A similar response pattern was observed for *M. chitwoodi*. The *A. dracunculus* EO caused 9.2% mortality after 24 h, which increased significantly to 89.0% at 48 h and 94.9% at 72 h. Conversely, *A. absinthium* showed minimal activity against this species, resulting in mortality rates of 1.1%, 4.3%, and 7.5% at the corresponding time intervals. The control groups exhibited no mortality throughout the experimental period (Table 6).

Based on these findings, dose–response assays were conducted using the *A. dracunculus* EO, as it was the only oil to achieve more than 90% mortality after 72 h. For *M. incognita*, the LC_50_ and LC_90_ values were determined as 0.8 and 2.1 µL/mL at 48 h, and 0.5 and 1.1 µL/mL at 72 h, respectively. EO required slightly higher concentrations to achieve similar lethality for *M. chitwoodi*. Dose–response analysis revealed LC_50_ and LC_90_ values of 1.1 and 3.5 µL/mL at 48 h, and 0.6 and 1.5 µL/mL at 72 h, respectively (Table 7).

### 3.7. Effect of Essential Oils on Egg Hatching of Meloidogyne spp.

As shown in Table 8, single-dose bioassays (2 µL/mL) were conducted to evaluate the effects of plant EOs on egg hatching of *M. incognita* and *M. chitwoodi*. Results indicated that *A. dracunculus* EO exhibited a low but measurable inhibitory effect on egg hatching. The inhibitory activity of this oil was more noticeable against *M. chitwoodi* (26.3%) than against *M. incognita* (17.3%). Conversely, *A. absinthium* EO resulted in negative inhibition values for both nematode species, −20.2% for *M. incognita* and −6.7% for *M. chitwoodi*. This suggests a stimulatory effect on the emergence of second-stage juveniles relative to the control group, rather than a suppressive one.

### 3.8. Sparse Partial Least Squares (sPLS) Results

**Figure 2:**
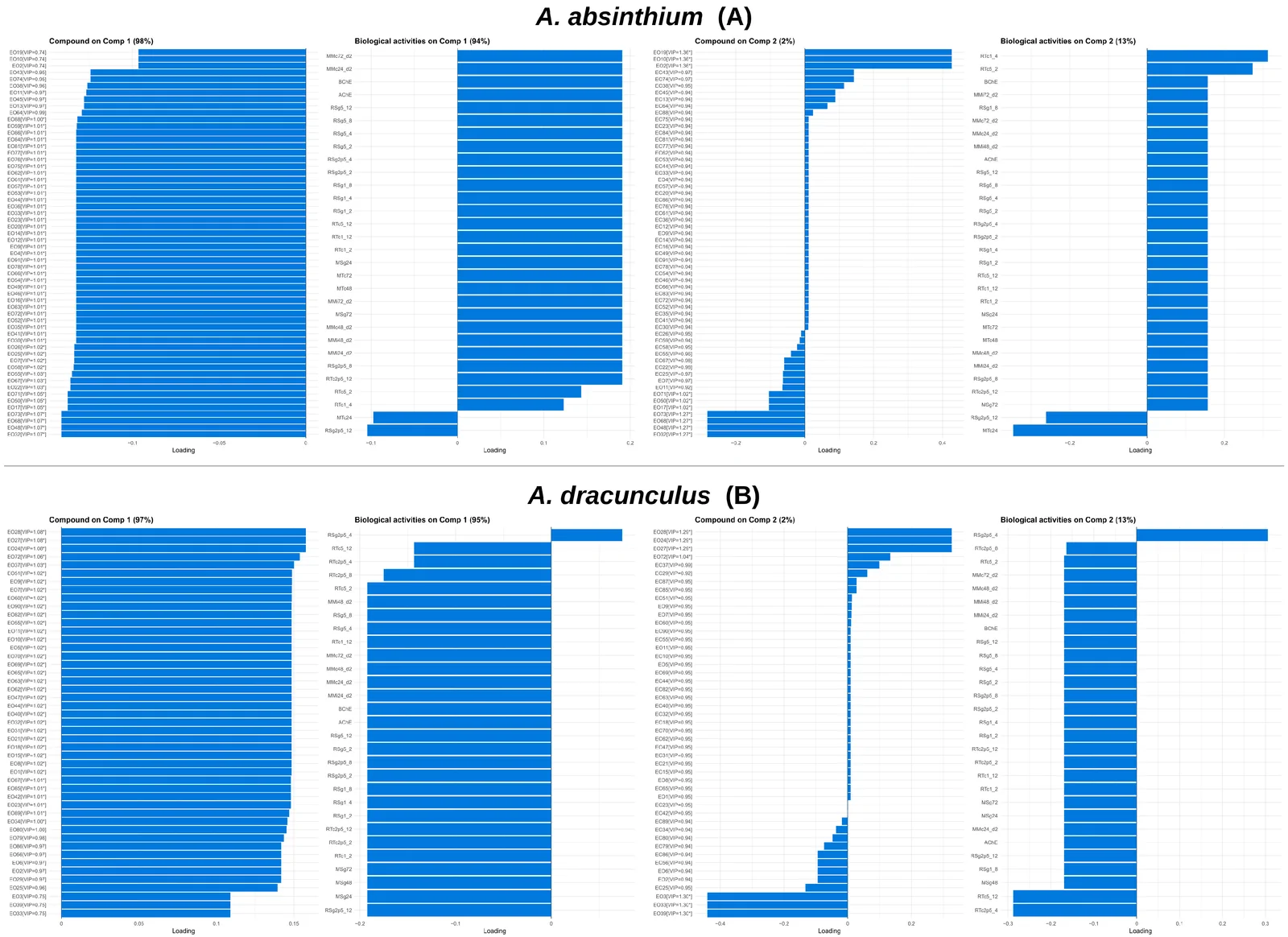
sPLS loading/VIP plots for the essential oils of *A. absinthium* (**A**) and *A. dracunculus* (**B**) (Comp1–Comp2). Left panels show X variables (EO constituents), and right panels show Y variables (AChE/BChE inhibition, contact toxicity, repellency, progeny suppression, and nematode-related endpoints). Variables with VIP ≥ 1.0 are marked with an asterisk (*).

**Figure 3:**
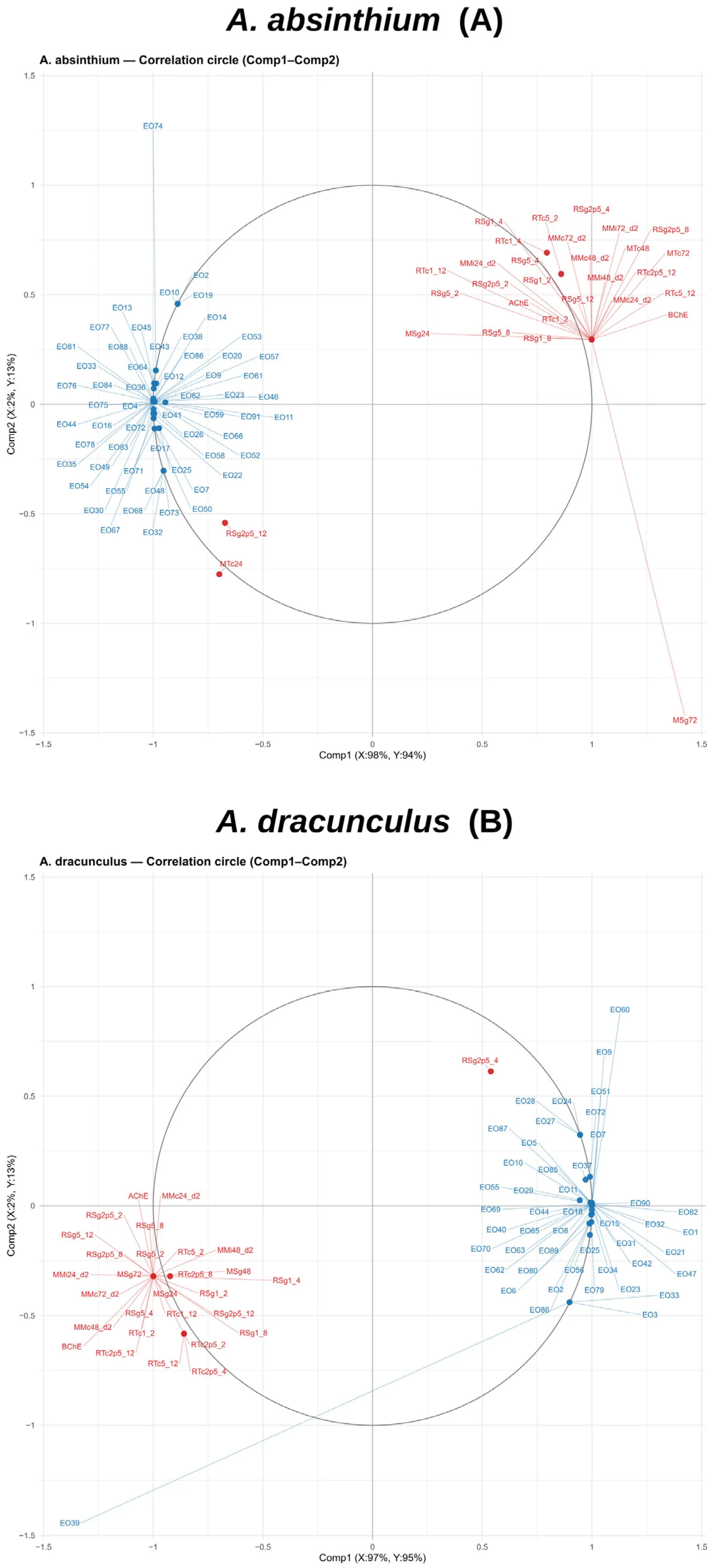
sPLS correlation circles for the essential oils of *A. absinthium* (**A**) and *A. dracunculus* (**B**) (Comp1–Comp2). For *A. absinthium*, Comp1 explains 98% of the X variance and 94% of the Y variance, whereas Comp2 explains 2% of the X variance and 13% of the Y variance. For *A. dracunculus*, Comp1 explains 97% of the X variance and 95% of the Y variance, whereas Comp2 explains 2% of the X variance and 13% of the Y variance. Variables closer to the unit circle are better represented. Variables oriented in the same direction indicate a positive association, whereas those oriented in opposite directions indicate a negative association.

**Figure 4:**
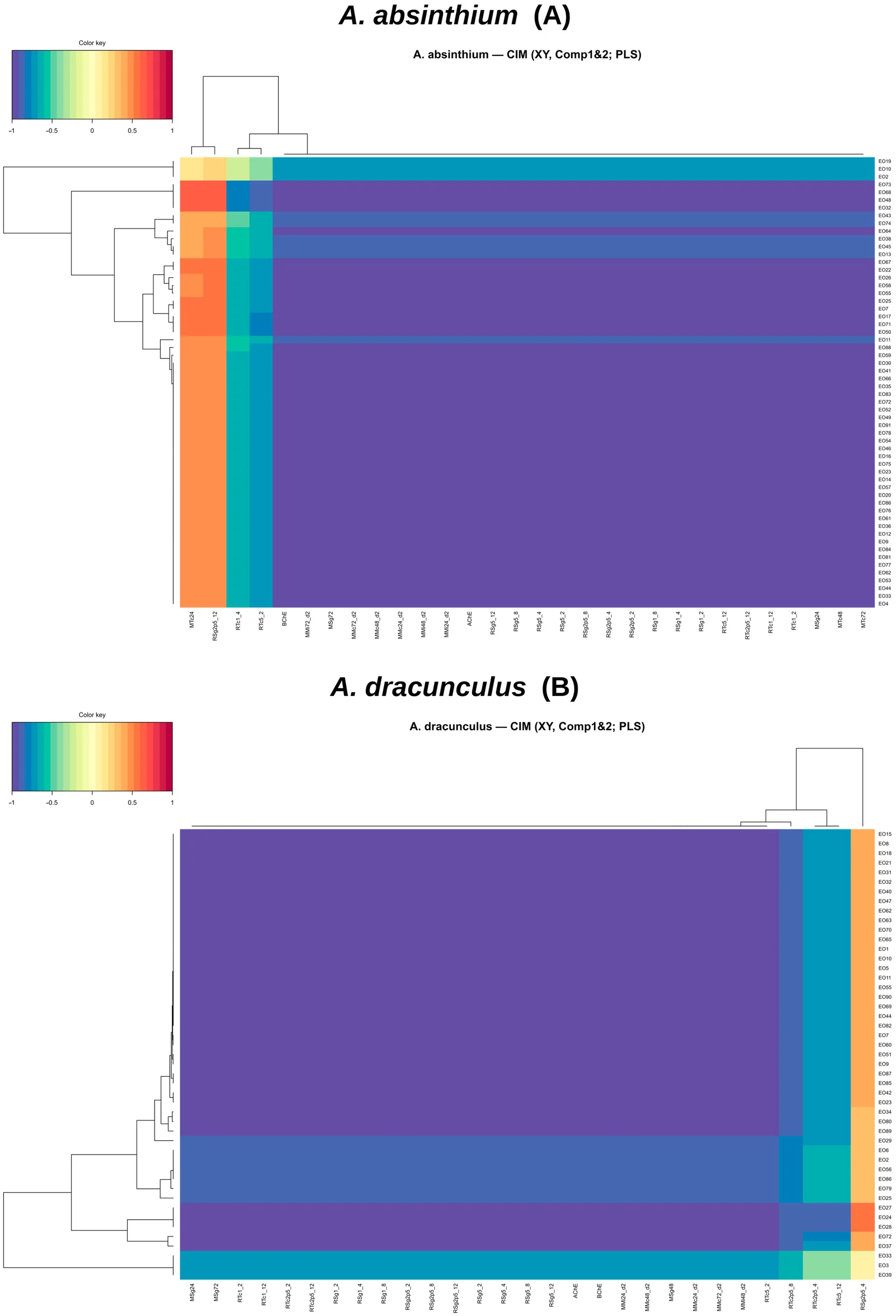
sPLS clustered image maps (CIM; X–Y correlation heat map based on Comp1 and Comp2) for the essential oils of *A. absinthium* (**A**) and *A. dracunculus* (**B**). The heat map displays correlation coefficients between EO constituents and biological parameters (−1 to +1); red indicates positive and blue indicates negative correlations, and hierarchical clustering groups variables with similar correlation profiles.

According to LOO-CV, the models showed high predictive ability (*A. absinthium* Q^2^ = 0.733; *A. dracunculus* Q^2^ = 0.793). The variance distribution indicated that Comp1 was dominant: in *A. absinthium*, Comp1 explained 98% of the X variance and 94% of the Y variance, whereas Comp2 explained 2% of X and 13% of Y; in *A. dracunculus*, Comp1 explained 97% of X and 95% of Y, whereas Comp2 explained 2% of X and 13% of Y. Loading/VIP profiles showed that AChE/BChE loaded strongly with the main biological pattern on Comp1, and EO constituents co-varying with this pattern included pulegone, methyleugenol, β-asarone, β-thujone, myrtenyl acetate, and β-phellandrene (Figure 2 and Figure 3). When the correlation circle and CIM were considered together, a substantial proportion of the biological variables clustered along Comp1, whereas MTc24, RTc1_4, RTc5_2, and RSg2p5_12 in *A. absinthium* and RSg2p5_4 in *A. dracunculus* deviated from the general pattern, with LOO-CV results indicating negative/unstable predictive performance for these measurements (Figure 4).

### 3.9. Molecular Docking Results

**Figure 5:**
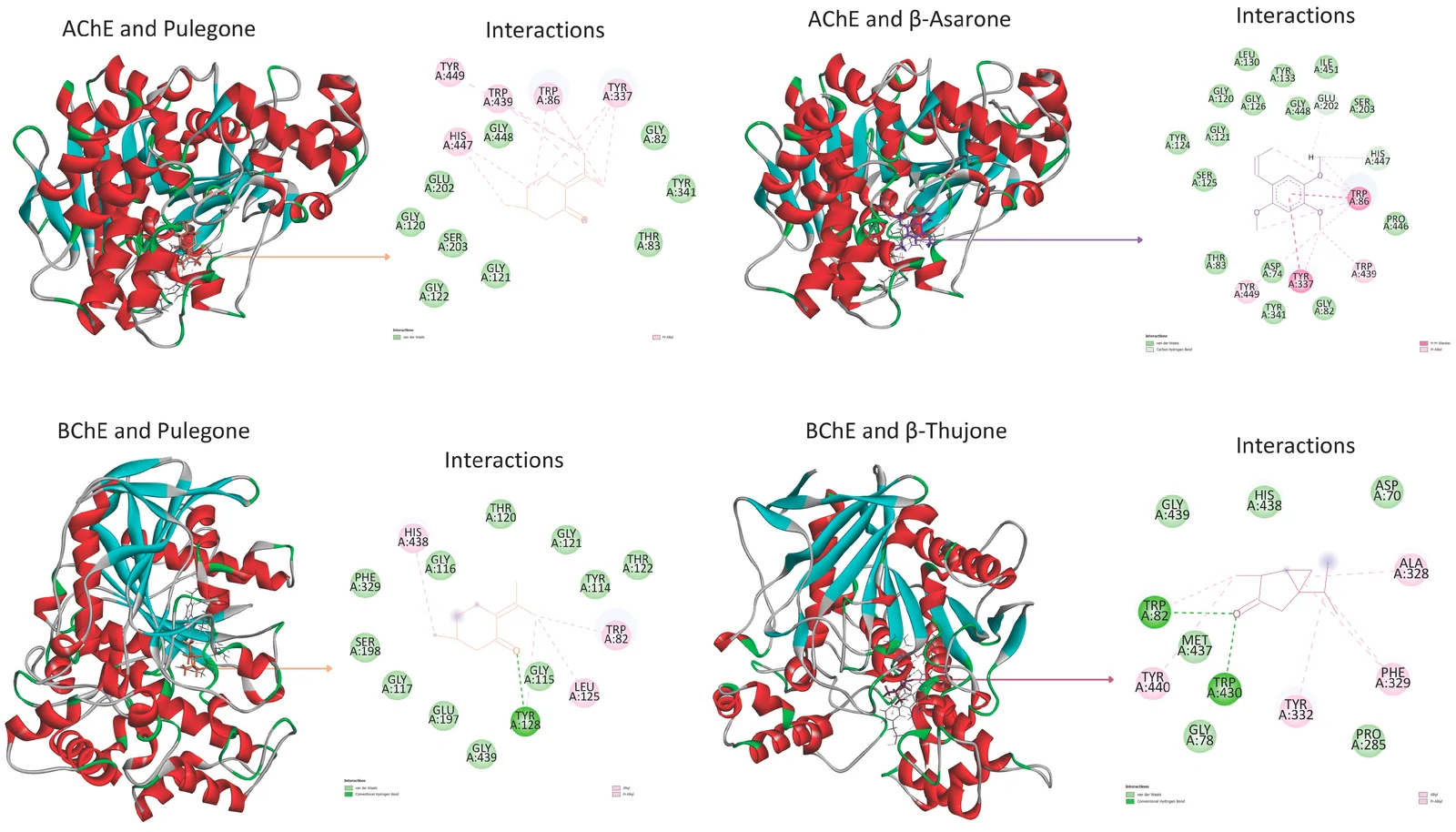
3D and 2D binding and interaction characteristics of selected ligands with acetylcholinesterase: AChE-pulegone and AChE-β-asarone (top; PDB: 7XN1) and BChE–pulegone and BChE-β-thujone (bottom; PDB: 4BDS).

The docked compounds (pulegone, methyleugenol, β-asarone, β-thujone, myrtenyl acetate, and β-phellandrene) are major constituents of the EOs of both species and were prioritized based on the PLS results, focusing on constituents co-varying with the experimental AChE/BChE inhibition profiles. Although all EO constituents yielded weaker docking scores than tacrine (AChE = −8.699; BChE = −7.941), within the selected set, the best (most negative) GlideScores were obtained for AChE with pulegone (−6.639) and β-asarone (−6.278), and for BChE with pulegone (−6.136) and β-thujone (−5.820) (Table 9). The interaction features of these top-ranked poses were analyzed and are summarized in the Supplementary Table S2 and Figure 5.

In the AChE–pulegone complex, the ligand is stabilized within the active-site gorge predominantly through hydrophobic/π-alkyl contacts. π-Alkyl interactions with Trp86 and Tyr337 are prominent, with additional hydrophobic/π contacts involving Trp439 and Tyr449. A van der Waals (vdW) contact with Ser203 near the catalytic center further supports accommodation of the pose in the vicinity of the active site. In the AChE-β-asarone complex, a more aromatic binding mode is observed, characterized by π-π/π-alkyl contacts with Trp86 and π-π/π-alkyl contacts with Tyr337. A vdW contact with Asp74 (PAS) and additional vdW contacts with Tyr124/Ser125 indicate that β-asarone can establish an extended interaction network along the gorge. In the BChE–pulegone complex, π-alkyl contacts with Trp82 and His438, together with a notable hydrogen bond to Tyr128 (2.92 Å), contribute to binding. Extensive vdW/hydrophobic contacts with residues along the gorge (e.g., Leu125, Gly115/116/117, Thr120/122, and Glu197) further support stable positioning of the ligand. In the BChE–β-thujone complex, a hydrogen bond with Trp82 (2.99 Å) is observed alongside π-alkyl contacts, and an additional hydrogen bond to Trp430 (2.92 Å) is identified. π-Alkyl interactions with Phe329 and hydrophobic/π contacts with Tyr332 and Tyr440 constitute the main interactions stabilizing the ligand within the active-site gorge.

## 4. Discussion

The GC–MS analyses revealed considerable qualitative and quantitative differences in the chemical profiles of *A. absinthium* and *A. dracunculus*. In *A. absinthium*, myrtenyl acetate was identified as the dominant constituent, whereas *A. dracunculus* was characterized by high levels of methyleugenol, β-asarone, and elemicin, which are primarily classified as phenylpropanoids. These interspecific differences were consistent with previous reports indicating that EO composition in *Artemisia* species is strongly influenced by genetic factors, environmental conditions such as altitude, soil type, and microclimate, plant phenology, and harvesting period [44,45]. From a bioactivity perspective, such compositional variability is highly relevant, as the chemical nature of the dominant compounds, whether monoterpenes, sesquiterpenes, or phenylpropanoids, largely determines volatility, lipophilicity, and mode of action against target pests. The predominance of phenylpropanoids in *A. dracunculus* could contribute to its insecticidal and nematicidal activity, as these compounds have been associated with interference in neuromuscular signaling, enzyme activity, and disruption of metabolic processes in both insects and nematodes [46,47]. Conversely, the abundance of myrtenyl acetate and β-thujone in *A. absinthium* could explain its strong acute insecticidal and repellent effects, as these monoterpenes are known to rapidly penetrate insect cuticles and affect the central nervous system [48,49].

The contact bioassays revealed distinct species-specific patterns in susceptibility. *A. absinthium* EO caused exceptionally high mortality in *S. granarius* but was ineffective against *T. castaneum*, while *A. dracunculus* EO exhibited moderate activity against *S. granarius* and no measurable effect on *T. castaneum*. This differential response is consistent with previous studies reporting species-specific variability in the efficacy of EOs against stored-product insects [50,51]. The limited activity against *T. castaneum* could be attributed to its physiological defenses, characterized by a thicker and more impermeable cuticle, which acts as a barrier to EO penetration. Furthermore, its efficient detoxification enzyme systems, such as cytochrome P450 monooxygenases and glutathione S-transferases, are capable of rapidly metabolizing toxic constituents before they reach their target sites [52,53]. In contrast, *S. granarius* is an internal grain feeder with a life cycle that often exposes adults to direct contact during handling, making them more vulnerable to fast-acting compounds. The high mortality caused by *A. absinthium* may also relate to synergistic interactions between its monoterpenes, increasing their ability to disrupt cellular membranes and block neuromuscular transmission.

Beyond acute toxicity, the behavioral responses observed in repellency assays indicated that both EOs acted as strong deterrents against *T. castaneum*, with effects being highly dependent on concentration and exposure time. At the highest concentration, *A. absinthium* maintained Class V repellency for up to 12 h, suggesting a notable residual efficacy of its active components. These results are consistent with previous work showing that *Artemisia*-derived monoterpenes such as β-thujone and camphor can evoke strong olfactory avoidance responses in coleopteran stored-product pests [15,32]. Against *S. granarius*, *A. absinthium* again outperformed *A. dracunculus*, which displayed more variable repellency, particularly at intermediate and low concentrations. The reduced repellency of *A. dracunculus* may be attributed to its phenylpropanoid-rich profile; these higher molecular weight and less volatile compounds disperse more slowly in the vapor phase and are generally less effective in eliciting rapid olfactory avoidance responses compared to monoterpene-dominated oils. This interpretation is aligned with established structure–activity relationships indicating that compound volatility and molecular size are key determinants of fumigant and repellent efficacy in stored-product insects [5]. Furthermore, the progressive decline in repellency over time likely reflects the loss of bioactive constituents through volatilization and chemical degradation processes [54,55].

Interestingly, reproductive inhibition patterns differed from acute toxicity trends. While *A. absinthium* caused higher adult mortality in *S. granarius* on contact, *A. dracunculus* more effectively suppressed F_1_ adult emergence in both pest species. This suggests that sublethal effects of *A. dracunculus*, potentially associated with phenylpropanoid constituents such as methyleugenol and β-asarone, may interfere with reproductive processes including oviposition and embryonic development. Phenylpropanoids are known to disrupt hormonal pathways, egg-laying behavior, and embryonic development in insects [56]. In contrast, the rapid knockdown effect of *A. absinthium* may kill adults before they lay eggs, but survivors could still reproduce effectively, leading to less suppression of progeny numbers. This pronounced reproductive suppression is further supported by the enzymatic findings, where *A. dracunculus* recorded significantly stronger inhibition of both AChE and BChE compared to *A. absinthium*. Inhibition of cholinesterase enzymes is associated with disruption of cholinergic signaling, which can impair motor activity, feeding behavior, and reproductive functions [5,54]. The dual inhibition of AChE and BChE may also suggest a broader neurotoxic profile, potentially affecting both neuronal and non-neuronal cholinergic pathways involved in insect development and reproduction.

In nematicidal assays, *A. dracunculus* attained nearly total mortality of *M. incognita* and *M. chitwoodi* within 72 h, whereas *A. absinthium* exhibited negligible efficacy. This observation is consistent with previous studies demonstrating the pronounced nematicidal activity of phenylpropanoid-rich EOs against plant-parasitic nematodes [24,57]. The combined presence of methyleugenol, β-asarone, and elemicin may contribute to enhanced bioactivity, potentially through additive or synergistic neurotoxic effects and improved penetration across the nematode cuticle. This is supported by evidence indicating that the nematicidal efficacy of EO constituents often results from synergistic interactions that facilitate better delivery across the cuticular barrier to the target receptors [58,59]. Moreover, the slightly greater susceptibility of *M. chitwoodi* compared to *M. incognita* may reflect interspecies biological variability within the *Meloidogyne* genus. Such variability may involve differences in cuticle surface characteristics and genetic differentiation that could influence physiological responses to foreign substances and detoxification pathways [60,61,62].

PLS analysis revealed a clear co-variation structure between EO chemical profiles and multiple biological outputs in both *Artemisia* species. The fact that Comp1 explains a large proportion of the variance and loads jointly with behavioral (repellency), lethal/sublethal (contact toxicity and progeny-related endpoints), and biochemical (AChE/BChE) responses indicates that EO-driven variation aligns with a common response pattern across a substantial part of the dataset. This interpretation is in line with studies reporting that EOs can influence not only the cholinergic system but also multiple pathways related to detoxification/antioxidant responses and metabolic processes [52]. Moreover, reviews highlighting that EO constituents may interact with insect nervous-system targets beyond AChE, such as GABAergic and octopaminergic signalling—support interpreting the terpene/phenylpropanoid pattern co-loading with AChE/BChE along Comp1 within a broader multi-target framework [63,64]. Consistently, studies linking *Artemisia* terpenes to antifeedant/repellent effects alongside AChE inhibition further support the observed co-variation in terpene/phenylpropanoid constituents with AChE/BChE along the Comp1 axis [65].

In contrast, negative or unstable $Q^{2}$ values for MTc24, RTc1_4, RTc5_2, RSg2p5_12 (in *A. absinthium*) and RSg2p5_4 (in *A. dracunculus*) are not unexpected. Repellency and early-time toxicity measures are sensitive to compound profiles, micro-environmental conditions, and short-term behavioral responses; therefore, a PLS model that summarizes EO composition primarily along a single linear axis can exhibit reduced predictive performance for these specific measurements. In addition, synergistic/antagonistic interactions among EO constituents and shifts in relative proportions can reduce linear co-variation for certain endpoints, consistent with reports of synergy/antagonism for EOs and binary combinations [66,67]. Finally, because LOO-CV leaves out one sample at a time, instability in $Q^{2}$ for particular endpoints can become more apparent under small-sample, multivariate settings [36]. Finally, focusing on variables with high VIP/loadings on Comp1 prioritizes not only the most abundant constituents but also those that co-vary with multiple biological responses, providing a rational basis for subsequent prioritization steps.

The docking results indicate that a limited set of major EO constituents, prioritized using the PLS analysis together with the experimental AChE/BChE inhibition data, can adopt consistent binding poses within the cholinesterase active-site gorge. Structural evidence highlights the importance of the Trp86 region in AChE and the Trp82 region in BChE for binding of tacrine/huprine-type inhibitors and points to conserved interactions in the vicinity of the catalytic histidine that contribute to pose stabilization [38]. In line with this, the top-ranked poses in our study were characterized by prominent π-based/hydrophobic contacts around Trp86/Tyr337 for AChE and around Trp82 for BChE.

Based on docking scores, pulegone/β-asarone ranked highest for AChE, whereas pulegone/β-thujone ranked highest for BChE. Nevertheless, docking captures binding at a single-compound/single-target level, whereas EO bioactivity is typically shaped by multi-component composition and multi-target pharmacology. EO constituents have been linked not only to AChE but also to GABAergic and octopaminergic targets in the insect nervous system, indicating that repellent/toxic effects are unlikely to be driven by a single enzyme [63,68,69]. In addition, synergistic or antagonistic effects have been reported for binary EO combinations, which may help explain cases where individual-compound docking rankings do not mirror in vitro outcomes [67]. Accordingly, our docking results should be viewed as structural support for the experimental AChE/BChE inhibition findings, while acknowledging that the overall EO bioactivity is likely mixture-dependent and multi-target in nature.

Taken together, these findings support the integration of *A. absinthium* and *A. dracunculus* EOs into sustainable pest management strategies, either as stand-alone botanical agents or as synergistic components within broader control programs. Future research should prioritize the development of advanced formulations to enhance stability, the evaluation of synergistic interactions with other bioactive compounds, and the validation of efficacy under field conditions to fully realize their potential in integrated pest and nematode management. Furthermore, the sPLS analysis revealed a robust co-variation structure between EO chemical profiles and the measured biological outputs, enabling a systematic prioritization of constituents that co-vary with AChE and BChE inhibition. Consistently, molecular docking identified pulegone as the top-ranked ligand for both enzymes, providing crucial structural validation for the experimental cholinesterase inhibition findings and highlighting its potential as a lead scaffold for the design of novel botanical pesticides.

## Figures and Tables

**Table 1:** The chemical composition of essential oils of *Artemisia absinthium* and *Artemisia dracunculus.*

No	RT	RI	RI *	Compounds Name	% Area		Formula
					*Artemisia absinthium*	*Artemisia dracunculus*	
1	6.55	913	921	*α*-Thujene	-	0.58	C_10_H_16_
2	6.82	924	926	*α*-Pinene	0.08	0.25	C_10_H_16_
3	7.88	933	929	*α*-Terpinyl propionate	-	0.07	C_13_H_22_O_2_
4	8.46	966	974	Phorbol	0.40	-	C_20_H_28_O_6_
5	9.23	979	989	*β*-Myrcene	-	0.22	C_10_H_16_
6	10.71	995	999	2-Carene	-	0.25	C_10_H_16_
7	11.29	952	1025	*β*-Phellandrene	0.15	15.91	C_10_H_16_
8	12.37	997	1083	Terpinolene	-	0.17	C_10_H_16_
9	13.01	1006	1022	*p*-Cymene	0.42	4.55	C_10_H_14_
10	13.26	1009	1008	3-Carene	0.09	0.19	C_10_H_16_
11	13.38	1015	1014	*α*-Terpinene	0.10	0.19	C_10_H_16_
12	15.72	1031	1026	Eucalyptol	0.24	-	C_10_H_18_O
13	17.56	1036	1026	D-Limonene	0.49	-	C_10_H_16_
14	19.23	1099	1095	*β*-Linalool	0.98	-	C_10_H_18_O
15	19.37	1104	1105	*α*-Thujone	-	0.17	C_10_H_16_O
16	20.24	1110	1115	*β*-Thujone	11.41	-	C_10_H_16_O
17	21.05	1130	1132	Calarene epoxide	0.32	-	C_15_H_24_O
18	21.79	1134	1136	cis-2-*p*-Menthen-1-ol	-	0.82	C_10_H_18_O
19	21.97	1142	1143	Isothujol	0.09	-	C_10_H_18_O
20	22.05	1149	1145	cis-Sabinol	1.40	-	C_10_H_16_O
21	22.69	1154	1146	Isopulegol	-	0.17	C_10_H_18_O
22	24.86	1155	1148	3-Thujanone	0.52	-	C_10_H_16_O
23	25.38	1180	1174	Terpinen-4-ol	0.59	4.88	C_10_H_18_O
24	26.57	1190	1183	*p*-Cymen-8-ol	-	0.11	C_10_H_14_O
25	26.66	1195	1186	*α*-Terpineol	0.15	0.27	C_10_H_18_O
26	26.80	1201	1191	Terpenediol I	0.55	-	C_10_H_18_O_2_
27	26.86	1204	1198	2-Pinen-10-ol	-	0.14	C_10_H_16_O
28	27.25	1207	1201	Estragole	-	0.11	C_10_H_12_O
29	27.54	1218	1209	Trans-Piperitol	-	0.10	C_10_H_18_O
30	28.62	1237	1235	Pulegone	8.50	-	C_10_H_16_O
31	29.59	1235	1236	Citronellol	-	0.20	C_10_H_20_O
32	31.23	1250	1244	5-Caranol	0.12	0.84	C_10_H_18_O
33	33.98	1254	1246	Grandlure II	0.17	0.08	C_10_H_18_O
34	34.84	1258	1259	1,4-dihydroxy-p-menth-2-ene	-	0.88	C_10_H_18_O_2_
35	35.28	1269	1263	trans-Chrysanthenyl acetate	1.04	-	C_12_H_18_O_2_
36	36.34	1275	1279	Phellandral	0.24	-	C_10_H_16_O
37	36.88	1287	1287	Isobornyl acetate	-	0.15	C_12_H_20_O_2_
38	37.41	1289	1288	R-Lavandulyl acetate	0.25	-	C_12_H_20_O_2_
39	38.29	1304	1296	D-Verbenone	-	0.09	C_10_H_14_O
40	38.84	1315	1310	Ascaridole	-	0.21	C_10_H_16_O_2_
41	43.96	1343	1326	Myrtenyl acetate	47.10	-	C_12_H_18_O_2_
42	45.69	1358	1354	Citronellol acetate	-	2.65	C_12_H_22_O_2_
43	47.69	1399	1405	*α*-Longipinene	0.29	-	C_15_H_24_
44	48.35	1408	1405	Methyleugenol	0.39	26.02	C_11_H_14_O_2_
45	48.77	1497	1410	Caryophyllene	0.48	-	C_15_H_24_
46	49.39	1463	1454	Neryl propionate	1.24	-	C_13_H_22_O_2_
47	49.76	1463	1462	Methylisoeugenol	-	0.40	C_11_H_14_O_2_
48	50.11	1459	1463	Dehydro-Aromadendrene	0.13	-	C_15_H_24_
49	50.82	1497	1490	*β*-Eudesmene	1.00	-	C_15_H_24_
50	51.31	1503	1500	*β*-Himachalene	0.33	-	C_15_H_24_
51	52.08	1509	1514	2,3,4-Trimethoxybenzyl alcohol	-	3.95	C_10_H_14_O_4_
52	52.47	1513	1515	2-ethyl-4-methyl-1,3-pentadienyl benzene	0.44	-	C_14_H_18_
53	52.95	1519	1515	Geranyl isobutyrate	0.39	-	C_14_H_24_O
54	53.13	1521	1516	2,2,7,7-Tetramethyltricyclo [6.2.1.0 (1,6)]undec-4-en-3-one	0.62	-	C_15_H_22_O
55	53.88	1523	1517	*α*-Curcumene	0.74	0.22	C_15_H_22_
56	54.01	1521	1519	Sesquicineole	-	0.25	C_15_H_26_O
57	54.96	1529	1527	(+)-Cuparene	1.38	-	C_15_H_22_
58	55.22	1536	1542	7-epi-cis-sesquisabinene hydrate	0.35	-	C_15_H_26_O
59	56.45	1539	1548	Diepicedrene-1-oxide	0.30	-	C_15_H_24_O
60	56.87	1559	1558	Elemicin	-	10.95	C_12_H_16_O_3_
61	57.17	1587	1580	Caryophyllene oxide	0.36	-	C_15_H_24_O
62	57.78	1597	1598	*β*-Elemenone	0.23	0.20	C_15_H_22_O
63	58.29	1598	1606	Methoxyeugenol	-	0.42	C_11_H_14_O_3_
64	58.43	1600	1606	trans-*α*-(Z)-Bergamotol	0.66	-	C_15_H_24_O
65	59.15	1601	1607	3,4,5-Trimethoxybenzaldehyde	-	2.76	C_10_H_12_O_4_
66	59.62	1608	1608	Neryl (S)-2-methylbutanoate	2.22	-	C_15_H_26_O_2_
67	60.69	1614	1618	Isoaromadendrene epoxide	0.54	-	C_15_H_24_O
68	61.63	1624	1630	*α*-Acorenol	0.14	-	C_15_H_26_O
69	62.04	1639	1641	*β*-Asarone	-	14.04	C_12_H_16_O_3_
70	63.25	1646	1642	tau-Muurolol	-	0.23	C_15_H_26_O
71	64.51	1654	1653	3-ethyl-5-(2-ethylbutyl) octadecane	0.34	-	C_26_H_54_
72	65.57	1677	1678	*β*-bisabolol	0.38	0.33	C_15_H_26_O
73	66.79	1694	1687	Nerolidyl acetate	0.13	-	C_17_H_28_O_2_
74	66.93	1696	1688	Cedr-8-en-13-ol	0.27	-	C_15_H_24_O
75	68.00	1705	1703	Juniper camphor	0.59	-	C_15_H_26_O
76	69.27	1733	1729	Murolan-3,9(11)-diene-10-peroxy	0.21	-	C_15_H_24_O_2_
77	70.49	1743	1735	trans-Nuciferol	0.23	-	C_15_H_22_O
78	71.13	1748	1740	Chamazulene	0.61	-	C_14_H_16_
79	72.94	1747	1746	Mandelic acid,3,4-dimethoxy,methyl ester	-	0.46	C_11_H_14_O_5_
80	73.24	1753	1751	3,4,5-trimethoxyphenyl-2-propanone	-	0.45	C_12_H_16_O_4_
81	73.28	1761	1760	cis-Lanceol	0.17	-	C_15_H_24_O
82	74.41	1790	1793	Coniferaldehyde methyl ether	-	1.02	C_11_H_12_O_3_
83	75.66	1797	1796	*β*-Santanol acetate	0.16	-	C_17_H_26_O
84	76.08	1836	1827	Hexahydrofarnesyl acetone	0.23	-	C_18_H_36_O
85	76.06	1877	1869	2,6-Dimethyl-2,6-octadien-8-yl acetate	-	0.30	C_12_H_20_O_2_
86	77.42	1883	1883	geranyl-α-terpinene	0.83	0.25	C_20_H_32_
87	78.70	1888	1888	Anhalinine	-	0.30	C_12_H_17_NO_3_
88	78.77	1920	1915	geranyl-*p*-cymene	3.07	-	C_20_H_30_
89	79.86	1971	1967	Fenoprofen	-	1.46	C_15_H_14_O_3_
90	80.76	2023	2016	*α*-Tetralone-5,6-dimethoxy-	-	0.44	C_12_H_14_O_3_
91	87.50	2272	2264	Larixol	0.62	-	C_20_H_34_O_2_
				Total	95.47	98.72	
				Monoterpene Hydrocarbons	23.40	26.98	
				Oxygenated Monoterpenes	2.89	11.11	
				Sesquiterpene Hydrocarbons	2.23	0.22	
				Oxygenated Sesquiterpenes	7.59	2.47	
				Others	59.36	57.92	

RI, retention index determined of a series of n-alkanes (C8–C30) on a HP-5MS; *, retention index of the literature used in identification on a HP-5MS Kovats’ RI non-polar column.

**Table 2:** Time-dependent contact toxicity of *Artemisia absinthium* and *A. dracunculus* essential oils (10%) against *Tribolium castaneum* and *Sitophilus granarius.*

Treatments	Mortality (%) ± SE ^a^					
	*Tribolium castaneum*			*Sitophilus granarius*		
	24 h	48 h	72 h	24 h	48 h	72 h
*Artemisia absinthium*	2.5 ± 2.1 b *	5.4 ± 1.0 b	5.4 ± 1.0 b	93.2 ± 0.1 a	95.0 ± 0.0 a	96.8 ± 0.5 a
*Artemisia dracunculus*	0.0 ± 0.0 b	0.0 ± 0.0 c	0.0 ± 0.0 c	66.3 ± 0.6 b	71.6 ± 0.8 b	73.5 ± 0.6 b
K-Obiol	92.3 ± 1.5 a	97.6 ± 0.9 a	100.0 ± 0.0 a	97.0 ± 1.2 a	100.0 ± 0.0 a	100.0 ± 0.0 a
Control	0.0 ± 0.0 b	0.0 ± 0.0 c	0.0 ± 0.0 c	0.0 ± 0.0 c	0.0 ± 0.0 c	0.0 ± 0.0 c
	F = 140.31 df: 3.19 *p* < 0.05	F = 209.99 df: 3.19 *p* < 0.05	F = 509.42 df: 3.19 *p* < 0.05	F = 190.0 df = 3.19 *p* < 0.05	F = 534.22 df = 3.19 *p* < 0.05	F = 408.17 df = 3.19 *p* < 0.05

^a^ SE: standard error of the mean. * Different letters following the means within the same column indicate statistically significant differences among treatments (ANOVA, *p* < 0.05, Tukey test).

**Table 3:** Repellent activity of *Artemisia absinthium* and *Artemisia dracunculus* essential oils against *Tribolium castaneum* at different concentrations and exposure times.

Essential Oils	Concentrations (%)	Repellency (%) ± StDev ^a^ (95% CI ^b^)			
		2 HAT ^c^	4 HAT	8 HAT	12 HAT
*Artemisia absinthium*	1.0	88.00 ± 8.37 a * (79.20; 96.80) Class V	92.00 ± 13.04 a (84.80; 99.20) Class V	100.0 ± 0.0 a (95.9; 104.1) Class V	46.00 ± 5.48 b (38.43; 53.57) Class III
	2.5	98.00 ± 4.47 a (89.20; 106.80) Class IV	98.00 ± 4.47 a (90.80; 105.20) Class V	96.00 ± 8.94 a (91.90; 100.10) Class V	56.00 ± 11.40 b (48.43; 63.57) Class III
	5.0	94.00 ± 8.94 a (85.20; 102.80) Class IV	100.0 ± 0.0 a (92.8; 107.2) Class V	100.0 ± 0.0 a (95.9; 104.1) Class V	90.00 ± 10.00 a (82.43; 97.57) Class V
*Artemisia dracunculus*	1.0	82.00 ± 14.83 a (73.20; 90.80) Class V	98.00 ± 4.47 a (90.80; 105.20) Class V	100.0 ± 0.0 a (95.9; 104.1) Class V	64.00 ± 11.40 b (56.43; 71.57) Class V
	2.5	86.00 ± 11.40 a (77.20; 94.80) Class V	94.00 ± 8.94 a (86.80; 101.20) Class V	96.00 ± 5.48 a (91.90; 100.10) Class V	84.00 ± 11.40 a (76.43; 91.57) Class V
	5.0	80.00 ± 12.25 a (71.20; 88.80) Class V	98.00 ± 4.47 a (90.80; 105.20) Class V	100.0 ± 0.0 a (95.9; 104.1) Class V	94.00 ± 8.94 a (86.43; 101.57) Class V

^a^ Standard deviations, ^b^ CI: Confidence Interval; ^c^ HAT: hour after treatment. * Different letters in the same row indicate statistically different from each other (ANOVA *p* < 0.05, Tukey test).

**Table 4:** Repellent activity of *Artemisia absinthium* and *Artemisia dracunculus* essential oils against *Sitophilus granarius* at different concentrations and exposure times.

Essential Oils	Concentrations (%)	Repellency (%) ± StDev ^a^ (95% CI ^b^)			
		2 HAT ^c^	4 HAT	8 HAT	12 HAT
*Artemisia absinthium*	1.0	62.00 ± 14.83 ab * (51.71; 72.29) Class IV	54.00 ± 8.94 ab (40.66; 67.34) Class III	38.00 ± 21.68 bc (29.24; 46.76) Class II	- -
	2.5	56.00 ± 13.42 ab (45.71; 66.29) Class III	68.00 ± 16.43 a (54.66; 81.34) Class IV	56.00 ± 8.94 b (47.24; 64.76) Class III	14.00 ± 15.17 cd (2.72; 25.28) Class I
	5.0	74.00 ± 15.17 a (63.71; 84.29) Class IV	72.00 ± 4.47 a (58.66; 85.34) Class IV	80.00 ± 7.07 a (71.24; 88.76) Class IV	50.00 ± 7.07 ab (38.72; 61.28) Class III
*Artemisia dracunculus*	1.0	38.00 ± 8.37 b (27.71; 48.29) Class II	30.00 ± 10.00 bc (16.66; 43.34) Class II	12.00 ± 8.37 d (3.24; 20.76) Class I	- -
	2.5	42.00 ± 8.37 b (31.71; 52.29) Class III	12.00 ± 14.8 3 c (−1.34; 25.34) Class I	32.00 ± 8.37 cd (23.24; 40.76) Class II	36.00 ± 18.17 bc (24.72; 47.28) Class I
	5.0	38.00 ± 16.43 b (27.71; 48.29) Class II	30.00 ± 17.32 bc (16.66; 43.34) Class II	56.00 ± 8.94 b (47.24; 64.76) Class III	66.00 ± 5.48 a (54.72; 77.28) Class III

^a^ Standard deviations, ^b^ CI: Confidence Interval, ^c^ HAT: hour after treatment. * Different letters in the same row indicate statistically different from each other (ANOVA *p* < 0.05, Tukey test).

**Table 5:** Inhibitory effects of plant essential oils on AChE and BChE enzyme activities.

Plants	Acetylcholinesterase	Butyrylcholinesterase
	Inhibition (%) + StDev ^a^	
*Artemisia absinthium*	14.6 ± 0.9	55.2 ± 0.6
*Artemisia dracunculus*	47.6 ± 0.7	67.7 ± 1.5

^a^ Standard deviations.

**Table 6:** The contact effect results of different plant essential oils against *Meloidogyne incognita* and *M. chitwoodi* at a single dose (2 µL/mL).

Species	Essential Oils	% Mortality ± StDev ^a^		
		24HAT ^b^	48HAT	72HAT
*Meloidogyne incognita*	*Artemisia dracunculus*	20.3 ± 0.5 a *	86.6 ± 0.1 a	100.0 ± 0.0 a
	*Artemisia absinthium*	1.2 ± 0.8 b	6.4 ± 0.8 b	11.4 ± 0.2 b
	Control	0.0 ± 0.0 c	0.0 ± 0.0 c	0.0 ± 0.0 c
		F = 101.38; df = 2.26; *p* < 0.01	F = 914.02; df = 2.26; *p* < 0.01	F = 503.26; df = 2.26; *p* < 0.01
*Meloidogyne chitwoodi*	*Artemisia dracunculus*	9.2 ± 0.2 a	89.0 ± 0.2 a	94.9 ± 0.2 a
	*Artemisia absinthium*	1.1 ± 0.2 b	4.3 ± 1.1 b	7.5 ± 0.1 b
	Control	0.0 ± 0.0 c	0.0 ± 0.1 c	0.1 ± 0.2 c
		F = 115.73; df = 2.26; *p* < 0.01	F = 2345.91; df = 2.26; *p* < 0.01	F = 2907.44; df = 2.26; *p* < 0.01

^a^ Standard deviations, ^b^ HAT: hour after treatment. * Different letters in the same row indicate statistically different from each other (ANOVA *p* < 0.05, Tukey test).

**Table 7:** Dose–response analysis results of the contact activity of *Artemisia dracunculus* essential oil against *Meloidogyne incognita* and *M. chitwoodi*.

Species	HAT ^a^	Slope ± SE ^b^	LC_50_ µL/mL (95% FL ^c^)	LC_90_ µL/mL (95% FL)
*Meloidogyne incognita*	48	3.0 ± 0.1	0.8 (0.4–1.2)	2.1 (1.4–12.3)
	72	4.3 ± 0.1	0.5 (0.5–0.6)	1.1 (0.9–1.4)
*Meloidogyne chitwoodi*	48	2.5 ± 0.1	1.1 (0.8–1.4)	3.5 (2.3–8.7)
	72	3.4 ± 0.1	0.6 (0.4–0.8)	1.5 (1.1–3.4)

^a^ HAT: hours after treatment, ^b^ SE: standard error, ^c^ FL: fiducial limit.

**Table 8:** Inhibitory and stimulatory effects (%) of plant essential oils (2 µL/mL) on the egg hatching of *Meloidogyne* species.

	*Meloidogyne incognita*	*Meloidogyne chitwoodi*
*Artemisia absinthium*	−20.2	−6.7
*Artemisia dracunculus*	17.3	26.3

**Table 9:** Docking results of major EO constituents against AChE (7XN1) and BChE (4BDS). (More negative scores indicate stronger predicted binding.) Values in bold indicate the two most negative docking scores for each enzyme.

Compound	AChE (7XN1) Docking Score (kcal/mol)	BChE (4BDS) Docking Score (kcal/mol)
Tacrine (reference)	−8.699	−7.941
Pulegone	**−6.639**	**−6.136**
β-Asarone	**−6.278**	−5.432
β-Phellandrene	−6.273	−5.731
Methyleugenol	−5.819	−5.231
β-Thujone	−5.557	**−5.820**
Myrtenyl acetate	−5.190	−5.710

## Data Availability

The original contributions presented in this study are included in the article. Further inquiries can be directed to the corresponding author.

## Supplementary Materials

The following supporting information can be downloaded at: https://www.mdpi.com/article/10.3390/insects17080837/s1, Table S1: Variable codes used in the sparse partial least squares (sPLS) analysis and their definitions. X block: individual essential-oil constituents identified by GC-MS. Y block: biological endpoints measured in the bioassays. Table S2: Interaction profiles of compounds with the highest binding scores in the active sites of AChE (7XN1) and BChE (4BDS).
