# Supplementary material for: Bioactivity of Artemisia dracunculus and A. absinthium Essential Oils Against Stored Product Insect Pests and Root-Knot Nematodes
Source: Insects. 2026 Aug 12;17(8):837. doi: 10.3390/insects17080837 (PMC13513081; doi:10.3390/insects17080837)
Supplement: Supplementary file 1 [file insects-17-00837-s001.zip › Supp_Table S2.pdf]

Table S2. Interaction profiles of compounds with the highest binding scores in the active sites of AChE (7XN1) and BChE (4BDS).

		**AChE**	
**Residue**	**Site**	**Pulegone**	**β-Asarone**
SER203	Triad	vdW	vdW
HIS447		π-Alkyl (4.87, 4.89)	C-H (3.08); π-Alkyl (4.15); vdW
GLY121	OxyH	vdW	vdW
GLY122		vdW	
TRP86	CAS	π-Alkyl (3.98, 4.29, 4.30, 4.55, 5.44)	π-π (3.65, 4.45); π-Alkyl (4.29, 4.49, 4.95, 5.04, 5.17)
TYR133			vdW
GLU202		vdW	C-H (2.29); vdW
TYR337		π-Alkyl (3.50, 4.18, 5.11)	π-π (4.90); π-Alkyl (3.32)
ASP74	PAS		vdW
TYR124			vdW
SER125			vdW
TYR341		vdW	vdW
GLY82	Omega	vdW	vdW
THR83		vdW	vdW
GLY448	BackDoor	vdW	vdW
TYR449		π-Alkyl (5.30)	π-Alkyl (5.25)
ILE451			vdW
GLY120	Other	vdW	vdW
GLY126			vdW
LEU130			vdW
TRP439		π-Alkyl (4.46, 4.60, 5.30)	π-Alkyl (4.44, 4.61)
PRO446			vdW
		**BChE**	
**Residue**	**Site**	**β-Thujone**	**Pulegone**
SER198	Triad		vdW
HIS438		vdW	π-Alkyl (4.28)
GLY116	OxyH		vdW
GLY117			vdW
TRP82	CBS	Hbond (2.99); π-Alkyl (3.62, 4.11)	π-Alkyl (5.10)
ASP70	PAS	vdW	
GLY78		vdW	
TYR332		π-Alkyl (4.72)	
LEU125	MidG		Alkyl (5.00)
TYR128			Hbond (2.92); π-Alkyl (5.50)
ALA328		Alkyl (3.82)	
PHE329		π-Alkyl (4.35, 4.93)	vdW
PRO285	Acyl	vdW	
TYR114	OxyLoop		vdW
GLY115			vdW
THR120			vdW
GLY121			vdW

THR122			vdW
GLU197			vdW
TRP430		Hbond (2.92)	
MET437	LowerG	vdW	
GLY439		vdW	vdW
TYR440		π-Alkyl (4.72)	

Site: Triad = catalytic triad; OxyH = oxyanion hole; CAS = catalytic anionic/choline-binding site; PAS = peripheral anionic site; CBS = choline-binding site; MidG = mid-gorge region; Acyl = acyl-binding pocket; BackDoor = back-door/gorge exit region; OxyLoop = oxyanion-loop vicinity; Other = other gorge-lining residues.
Interactions: vdW (van der Waals); Hbond (conventional hydrogen bond); C–H (carbon–hydrogen bond); π–π (π–π stacking); π-Alkyl (π–alkyl contact); Alkyl (hydrophobic alkyl contact).
Distances: Å and shown in parentheses; if multiple, they are separated by commas.
